# The learning experiences of dyslexic medical students during the COVID-19 pandemic: a phenomenological study

**DOI:** 10.1007/s10459-021-10074-7

**Published:** 2021-09-17

**Authors:** Sebastian C. K. Shaw, Laura R. Hennessy, John L. Anderson

**Affiliations:** 1 Department of Medical Education, Brighton and Sussex Medical School, Brighton, East Sussex UK; 2 Weston General Hospital, University Hospitals Bristol and Weston NHS Trust, Weston-Super-Mare, UK

**Keywords:** Dyslexia, Medical students, Medical school, Medical education, Qualitative, Coronavirus, COVID-19

## Abstract

Dyslexia is a Specific Learning Difficulty that impacts on reading and writing abilities. During the COVID-19 pandemic, medical schools have been forced to undertake distance learning and assessment. The wider literature suggested that e-learning might pose additional challenges for dyslexic students. Here we explore their overall experiences of learning/studying during this time in a phenomenological study. Five medical students were interviewed in depth and the audio-recordings were transcribed verbatim. Transcripts then underwent an interpretive phenomenological analysis. Our results highlighted a largely positive experience, with an improved culture of togetherness, freedom and sense of control. They also revealed issues with a lack of clinical exposure, potential negative impacts on ranking positions for those with dyslexia, and possible cheating in exams. There are some surprising results—in particular the positive responses to how remote learning was delivered. These seemed to put our participants more on a par with their non-dyslexic colleagues—except in some examinations. It is our hope that medical educators may resist a return to ‘the way things have always been done’ when the pandemic has resolved, and by doing so, continue to foster this new, positive culture and paradigm shift.

## Introduction

Disability is not a flaw, an individual tragedy nor a whispered recognition of another’s embodied failing or a shameful family truth. Disability is a matter of public discourse and international disgrace, exemplified in the continued exclusion of impaired children from mainstream schools… the segregation of disabled adults from employment contexts… and the denial of access to basic human rights as a consequence of reducing welfare and essential services (Goodley et al., 2019).

The critical disability studies movement was willed into existence by the activism work of disabled individuals in the 1970s (Reaume, 2014). This movement brings together academics from a variety of background fields (Reaume, 2014), including medical education, with the shared goal of shedding light on social inequity and the experiences of disabled people (Goodley et al., 2019). In doing so, it scrutinises the marginalisation and oppression of these societal groups (Goodley et al., 2019). In that sense, the critical disability studies movement strives to promote social justice. The UK Equality Act (2010) defines a disability as “a physical or a mental impairment” which “has a substantial and long-term adverse effect on a person’s ability to carry out normal day-to-day activities” (Great Britain, 2010). However, from a sociological perspective, there can be differing views on this. A medical approach views a disability as a series of weaknesses within an individual, as evidenced in the aforementioned legal description. Such views promote discussion of symptoms or defects and treatments (Shakespeare & Watson, 2015). This approach has, however, been criticised within the disability studies movement for overlooking associated strengths and social or environmental impacts on day-to-day functioning (Shakespeare & Watson, 2015). Therein lies the birth of the social model of disability (Oliver, 2013). This views disabilities from a different perspective. The social model argues that it is in fact societal and environmental factors that cause disablement in individuals with impairments (Oliver, 2013).This takes the emphasis off of biological issues within the individual, and instead scrutinises disability as a sociocultural issue (Walker & Shaw, 2018). In the case of neurodevelopmental conditions, the neurodiversity movement can provide another theoretical lens. This takes a step beyond the social model of disability to view these as differences—an aspect of normal human diversity—rather than considering individuals to have overt underlying impairments. This has been both applauded and critiqued in the wider literature (Clouder et al., 2020; Nelson, 2020).

Socially grounded views of disability, such as those discussed, have paved the way to increased consideration of inclusivity and accessibility in education. As such, widening access has been a central focus of Higher Education (HE) over recent years. For example, within the United Kingdom (UK), the Government provides widening access targets for HE institutions (Connell-Smith & Hubble, 2018). This same drive to widen access also exists within medical education (British Medical Association 2020). One aspect of this includes the realisation of equitable admission policies for disabled individuals into HE (Medical Schools Council, 2018). A recent report found that UK medical schools, following a doubling in the amount of disabled entrants, are now admitting a similar proportion of disabled students to general HE (10%) (Medical Schools Council, 2018). This is suggestive of positive change, at least at the stage of admissions. Specific Learning Difficulties (SpLDs), a cluster of diagnoses that includes dyspraxia, dysgraphia, dyslexia, dyscalculia and attention deficit hyperactivity disorder, traditionally falls within the category of disabilities (Musto, 2013; Walker & Shaw, 2018). These refer to differences within specific areas of learning (e.g. reading or writing) rather than to impairments of individuals’ overall cognitive abilities (Musto, 2013). In this study, we specifically consider *dyslexia*—a SpLD that impacts reading and writing. In keeping with other SpLDs, it does not impact intelligence (British Dyslexia Association n.d.). Dyslexia effects an estimated 10% of the UK population (Dyslexia International n.d.), and there have been no published prevalence data in medical education in over a decade—during which time, as aforementioned, there has been much in the way of widening access initiatives. It is therefore plausible to assume that we may be seeing increasing numbers of dyslexic medical students.

Various aspects of medical education may challenge dyslexic students and risk overwhelming pre-existing coping strategies. Such challenges may be further exacerbated by the Coronavirus Disease 2019 (COVID-19) pandemic. COVID-19 is the most significant global health crisis of our generation (Ahmed et al., 2020) and has subsequently had significant impacts on the mental wellbeing of healthcare practitioners (Shaw, 2020). Increasing workloads and staff shortages have the potential to induce both hopeless and helpless states in junior doctors (Shaw, 2020). Its impacts have also drastically altered the way in which we train healthcare students, such as the suspension of in-person teaching (Ng & Or, 2020). HE institutions have shifted their emphasis to online delivery of their classes (Ng & Or, 2020). In essence, the COVID-19 pandemic has triggered a shift in educational practice that has created a quasi-experimental situation not otherwise possible. Students have been undertaking large elements of their medical education remotely through e-learning. This has afforded us an opportunity to access students’ reactions to learning environments which would not have arisen otherwise. Whilst the shift to remote, e-learning is in keeping with various worldwide guidelines regarding social distancing, it has the potential to introduce some issues for learners—particularly for those with SpLDs. It has been argued that e-learning (also known as online learning) may potentially disadvantage dyslexic learners by creating additional barriers through its emphasis on the written word (Woodfine et al., 2008). It has also been argued that e-learning may be more time-consuming and may require more effort for these students compared to traditional teaching approaches (Alsobhi et al., 2015). No research has considered the impacts of dyslexia on e-learning, or dyslexic students’ experiences with regards to e-learning (Alsobhi et al., 2015). These students may be experiencing new and unfamiliar barriers. In keeping with the social model of disability, this new learning environment could, in theory, disadvantage dyslexic students. A previous survey of dyslexic junior doctors in the UK found that 70% reported slow speeds of reading, 54% reported struggling to articulate thoughts accurately in writing, 36% reported difficulty reading from screens, and 35% reported taking longer than others to grasp concepts (Anderson & Shaw, 2020). At face value, e-learning therefore has the potential to increase the workload of dyslexic students. Past research has also shown that dyslexic medical students can carry a heavy emotional burden (Shaw & Anderson, 2018b). Therefore, the added workload associated with e-learning may put these students at greater risk of burnout—particularly in the context of the COVID-19 pandemic, which, in itself, is likely to provoke negative emotional responses and strain (Shaw, 2020).

Evidence suggests that dyslexic students in higher education already experience a lack of understanding from their tutors (Madriaga, 2007). This lack of understanding is also reflected in the experiences of dyslexic medical students, albeit less specific in nature (Anderson & Shaw, 2020; Shaw & Anderson, 2018b). Our previous work (SS and JA) has found that dyslexic medical students reported a general lack of understanding of dyslexia and its impacts from those around them (Shaw & Anderson, 2018b). Furthermore, 16% reported being bullied or ridiculed by medical school teaching staff, with 30% reporting bullying/ridicule from their clinical teachers (Anderson & Shaw, 2020). This may act to marginalise such students, further reducing their sense of understanding and acceptance. Given this, whilst it is important that we consider the added impacts of e-learning, it is also important that we understand the potential wider impacts of remote learning. It is also vital that we consider how to best support dyslexic students through the current situation in order to prevent our teaching inadvertently disabling them. In order to best consider this, we should explore the experiences of dyslexic medical students in relation to this. We were unable to locate any studies of the experiences of dyslexic medical students in relation to COVID-19. Our main research question was: “What are the learning experiences of dyslexic medical students during the COVID-19 pandemic?” Our project aimed to explore this in relation to e-learning, self-study, clinical experiences (or lack thereof) and any emotional impact. Through exploring these important areas, we aimed to shed light on the current situation and, subsequently, consider learning or support adaptations.

### Author backgrounds

Within qualitative research it is important to provide readers with an overview of ourselves as the researchers/authors—both in terms of our experiences and our strengths. This may allow readers to better understand our roles as both the data gathering and data analysis instruments within the research.

SS is an Honorary Clinical Lecturer at Brighton and Sussex Medical School. He has a strong background in qualitative research—in particular with autoethnographic and phenomenological studies. As part of his role at the medical school he teaches postgraduate students in obtaining informed consent and in the analysis of qualitative data. He has also previously taught qualitative interview skills at the medical school. He has a special interest in neurodiversity in medical education and, alongside JA, has published widely in this area. Some of his previous work on dyslexia includes (Anderson & Shaw, 2020; Hennessy et al., 2020; Shaw, 2018; Shaw & Anderson, 2017, 2018b; Shaw et al., 2016, 2017, 2018, 2019) and on dyspraxia includes (Walker et al., 2018, 2020, 2021). He and JA have also previously published educational guidance on undertaking and publishing phenomenological research in medical education (Shaw & Anderson, 2018a). His interest in this particular area stems from the fact that he is dyslexic himself.

LH is a Foundation Year Two Doctor at Western General Hospital. She has a particular interest in dyslexia within medical education and has previously undertaken research in this area alongside SS and JA. Her relevant publications include (Hennessy et al., 2020; Shaw et al., 2019). She has been trained by SS in obtaining informed consent, in interview skills and in analysing qualitative data. She is also dyslexic.

JA is a Principal Lecturer at Brighton and Sussex Medical School. He has had extensive experience with both qualitative and quantitative research since the 1970s. He and SS have been undertaking in a series of research into dyslexia in medical education over the past seven years.

## Methods

This is an interpretive phenomenological study, within an interpretivist paradigm. We adopted a similar approach to our previous phenomenological work exploring the experiences of dyslexic medical students (Shaw & Anderson, 2018b) and the experiences of dyspraxic foundation doctors (Walker et al., 2021).

### Philosophical and methodological basis

Phenomenology refers to the qualitative study of people’s “lived experiences”. We adopted an Interpretive Phenomenological Approach (IPA), which stems from the work of Martin Heidegger (Mackey, 2005). IPA takes a step further than descriptive phenomenology, by striving to seek meaning and understanding in people’s experiences (Lopez & Willis, 2004). These meanings may not even be known to the participants (Shaw & Anderson, 2018a). IPA also allows researchers to capitalise on any prior experiences of their own in the design, conduct and analysis of studies—declaring and embracing them as part of its interpretive approach (Shaw & Anderson, 2018a). Lopez and Willis discuss this further, outlining the inherent researcher-participant intersubjectivity involved in the approach and its associated strengths (Lopez & Willis, 2004)—thereby placing this research approach truly within an interpretivist paradigm (Shaw & Anderson, 2018a). “When one interacts with another in an act of understanding and getting to know each other, it is based on a personal horizon of experiences and meanings. This means that the act of interpretation is always bounded by the separate and intersecting horizons of human beings: both researcher and participant” (Lopez & Willis, 2004). “This process involves actively engaging in a hermeneutic process, with the researcher reflecting and acknowledging their fore-structures (previous experiences and pre-conceptions)” (Holland, 2014). The interpretive nature of this approach was therefore particularly well suited to our study, given the near-insider nature of our research team—both SS and LH are dyslexic doctors.

#### Ethical review

The Brighton and Sussex Medical School Research Governance and Ethics Committee approved this study. In addition to this, we actively engaged in a relational ethical approach throughout. In previous work, SS has elaborated as follows:

At its core, qualitative research is all about interactions with the personal realities of its participants, be that their experiences, beliefs, or cultures. Sometimes these interactions may be quite intimate in nature, and may leave participants, or even the researcher feeling vulnerable... Never underestimate the power of spoken, or in this case written words on the influence of emotional wellbeing. Therefore, qualitative research has the capacity to cause a great deal of emotional and reputational harm to both researchers and participants… We must remain true to our participants, our research questions, and ourselves at all stages. And we must ask ourselves: is this the right thing to do? Is this the right thing to write? And have I considered the wider implications of what is done or written? (Shaw, 2019).

In practical terms, we did this in several ways. For example, where participants mentioned specific third parties, we ensured their details were omitted from transcripts and report. Where participants reported something that we felt may identify themselves, we also omitted these from our report. Finally, where participants reported anything that may hold fitness to practice concerns for themselves or others, we discussed these issues in-depth as a team to consider the most ethically appropriate actions to take. Through adopting such strategies, we aimed to safeguard the wellbeing and anonymity of our participants to the best of our ability, whilst also balancing the important ethical tenet of social justice.

### Inclusion/exclusion criteria

Participants had to meet all of the following inclusion criteria to be eligible:

- Be medical students within the included medical school.
- Be in year 1–4 of their medical degree.
- Have a diagnosis of dyslexia.

Fifth (final) year students were not included due to their time being given to clinical commitments on the front line of the National Health Service in the COVID-19 pandemic. We did not wish to distract from these duties.

### Recruitment

Emails were sent to all year 1–4 medical students in a single medical school in the South of England. Interested individuals were asked to self-identify and contact us by email if they were interested in taking part. A Participant Information Sheet and Consent Form were then emailed to interested individuals. Those still wishing to participate were invited to give us their informed consent verbally over Microsoft Teams. This was audio-recorded and then stored securely on our university server.

### Data collection

Our data collection method of choice was loosely structured, one-to-one interviews. An interview topic guide was generated by SS and LH in an iterative process, making use of their insider experiences. This topic guide explored experiences in relation to:

- self-study.
- E-learning.
- clinical learning (if applicable).
- Emotional experiences in relation to learning/studying.

Interviews were conducted by SS and LH. These lasted approximately forty-five minutes each and took place over the Microsoft Teams video conferencing platform. Interviews were audio-recorded. Recordings were immediately transferred to our university server to be stored securely.

### Data analysis

Interview audio-recordings were transcribed verbatim by SS and LH. Transcripts then underwent an interpretive phenomenological analysis using the approach of Pietkiewicz & Smith (Pietkiewicz & Smith, 2014). First, the authors immersed themselves in the data. During this process, they made notes on the transcripts. As Pietkiewicz and Smith (2014) discuss, “it is useful to highlight distinctive phrases and emotional responses” at this stage. Notes and transcripts were then reviewed to identify initial emergent themes. These emergent themes were then scrutinised to identify relationships between them—leading to the generation of analytical theme clusters. Finally, these theme clusters were compared back to the original transcripts to ensure that they were representative of the data. Disagreements were discussed and re-analysed until the final analysis was agreed upon. This was, once again, an iterative process.

## Results

Five people participated—two males and three females. They spanned years 1–3 of medical school. Three were from pre-clinical years and two were from clinical years. An overview of our theme clusters is presented in Table 1.

**Table 1:** Themes and sub-themes

Themes	Sub-themes
Taking control of their own education	Enjoyment of education
	Technology-enhanced learning
	Having their own space and routines
	Freedom to review materials later
	Reduction in pressure
A paradigm shift in education	Embracing inclusive teaching, assessment and support
	Ineffectiveness of lectures and traditional teaching
	Desire for the new learning approaches to continue
Changing social dynamics	Kindness, unity, and acceptance
	Growing apart
	Seeing others—a frame of reference
Worries and wonderings	Venturing into the unknown
	Lack of clinical exposure
	Clinical assessments, ranking and cheating
	Technological issues

### Theme 1: taking control of their own education

#### Subtheme 1a: enjoyment of education

All participants expressed a sense of taking back control of their education—from self-study to self-timetabling. They had enjoyed this period of their medical studies. For example, P4 explained that “I am really enjoying it… ‘cos I can’t sit still in lectures—I get really, really bored… I, umm, have really enjoyed being able to do a lot of it online and being able to pause and make a cup of tea, for example, and then carry on.” P3 felt particularly strongly that this had been the best period of his degree: “I’ve enjoyed it actually much more… This new layout (distance learning) has felt like it’s been my optimal thing.” This positive experience was shared with P2, who felt that “it was a much better learning experience—less stressful, and actually much more fun.”

#### Subtheme 1b: technology-enhanced learning

Most were grateful for the accessibility of the technologies used to deliver teaching. P1 found that “when I couldn’t hear… [or] couldn’t understand something properly, I would slow the speed of the lecture down.” Furthermore, P5 found that the use of an online question bank was “amazing”, and that is formed “about 90% of my revision.” Others made use of electronic flashcard software—especially where these could be available on multiple platforms. “You can also have the app on your phone as well, so you can just do a couple of minutes of questions” (P2). Some felt that online videos were also very helpful. For example, P2 explained that “The [anatomy] lectures weren’t particularly good, because they were using mainly models from plaster models, and diagrams which weren’t accurate enough to real life body parts. So… I was using videos… I’ve found those videos really useful.” Video communication technology was also praised for facilitating the learning experience during lockdown. P5 explained how he and a colleague “constantly revise over Facetime… He’ll do, say, one half of the diseases, I’ll do another half. And then we’ve got to teach the other person what we’ve learned that day.”

#### Subtheme 1c: having their own space and routines

Homeworking granted participants greater flexibility and control over their learning. They were able to develop their own learning-related idiosyncrasies, and to feel comfortable in their chosen environments. For example, P2 found that “I can go through things at my pace… I can absorb more information and do work much more quickly than I used to—compared to in the medical school. So, yeah. I think that the lockdown did benefit me a lot.” They felt that this allowed them to flourish in their studies. P4 pointed out that “being able to structure my day how I want it structured… I feel that maybe I’ve been more… productive.” This relaxed state of mind was also expressed with regards to exams. P3 explained that “being at home, where I’ve, like, been sat at this desk studying most of the time… This is like my own little environment… So, actually, sitting here and doing an online test… Just having my own space… Doing it online has been brilliant.”

#### Subtheme 1d: freedom to review materials later

Homeworking introduced a degree of freedom. Participants saw this is a great improvement from traditional, lecture-based medical education. For example, P2 felt that “back at home I can just take my time, and re-visit the material I couldn’t understand before, and then merge things together… So that really did help me understand a lot better the material.” With this freedom came a sense of security. Participants no longer felt panicked about being disadvantaged by missing teaching session. P3 explained that “if something goes wrong on someone’s end, it’s easy to just be like ‘oh, I’ll just watch it later’… because it’s all recorded and stuff.” Furthermore, P5 highlighted that “being able to go through the recordings, where I can slow things down—I can pause, so I can catch up with my writing, and I can go over things multiple times—has actually made it a lot easier for me.” However, this freedom came at a cost for some—studying ate into their personal time. This was highlighted by P1, who found that “it has taken a lot more time… I don’t think there was day where I totally didn’t do anything. I think I always so of carried over 1 lecture to the next day… I just used my weekends.” Despite this, all reported it to be a more useful and positive experience overall.

#### Subtheme 1e: reduction in pressure

Due to the senses of security and control, participants felt a great reduction in pressure and associated stress. For example, they experienced fewer time pressures. P1 found that “when you are at home, there have been times where I have actually found it easier, because I can go at my pace and I can take 2 h for something that actually, during a lecture, we would have only had an hour for.” P3 strongly agreed with this, feeling that it is a great improvement from medical school life before COVID-19. He explained that “I found pre-lockdown quite frustrating—having to sort of travel in and spend an hour for a 45-min lecture—have a break and then… I found that kind of an inefficient use of time—quite frustrating when I know it takes me a bit longer.” This sense of reduced pressure also extended to exams/assessments—enabling them to become learning experiences in themselves. P1 found that “with [the anatomy viva] moving online it was so much better to be able to have more time and to write it and to not to have that pressured environment… I learnt so much more from that exam” (P1).

### Theme 2: a paradigm shift in education

#### Subtheme 2a: embracing inclusive teaching, assessment and support

All participants were wholeheartedly grateful to their medical school for its efforts to embrace *inclusivity* in its distance teaching approaches. There was a general feeling that their medical school was open to supporting diverse students however they could. P5 explained that “I don’t know if it’s (support needs) something that I’ve talked to them (the medical school) specifically about. But I know that I always could if I wanted to.” More specifically, two participants found the addition of manually typed captions to videoed presentations to be vitally important to their learning and understanding. For example, P2 said that “they decided they would write down what the lecturers are saying and put it in writing on the screen… That really helped.” P4 also explained that “they did record most of [the online lectures], so you didn’t have to attend… if you felt like you couldn’t.”

The inclusive approaches that participants valued also extended to their exams. P3 explained that “the [exam] interface was really nice. It was really clear. You could go and modify it and change it to make it friendly colours and increase the size of the text, and you could highlight bits in the question… and exclude answers.” P5 felt the same way: “It has actually been quite nice… for the exam software, you can change the colour and the size as well… The online exam also allows you to cross out questions. So, where it’s multiple choice… You could cross out the ones that you definitely knew weren’t the answer. That was the main thing I was really worried about… So, that was really good.”

Despite the aforementioned thankfulness for inclusive changes, participants recognised that there were no perfect solutions. “I definitely don’t think that there’s a scenario in which everybody comes out of this happy with COVID. Because, if you’re being fair to me, you’re putting somebody else at a disadvantage” (P5).

#### Subtheme 2b: ineffectiveness of lectures and traditional teaching

Most participants reported negative experiences relating to traditional, face-to-face lectures. “I’ve always found that lectures aren’t very helpful… I can’t keep up…” (P3). This was mirrored by P2, who said “if it is a 3-h lecture, I kind of switch off after 1 h.” These difficulties led to participants rebelling. For example, P4 explained that “I was quite bad during [pre-clinical years] and didn’t really attend lectures.” P3 explained that “I have brought it up with people before [at my medical school] and said there’s loads of evidence and studies on it saying that it’s not effective learning—it’s just about delivery of information.”

#### Subtheme 2c: desire for the new learning approaches to continue

Participants expressed a desire for their education to maintain these changes upon resolution of the pandemic. P3 said that “for me, I would like all the lectures to be done online and have module tutorials face-to-face. Or, maybe twice a week you go in and have a small-group session—and then all the other stuff is self-directed learning at home.” P2 felt that “rather than [doing things] strictly [on a] face-to-face or one-to-one basis, we can actually do things online or virtually.” P5 also felt that the online resources used during this time should continue to be created afterwards, due to their improved educational quality: “I think everybody’s vocalised that, even post-COVID, we’d like to continue getting those.”

### Theme 3: changing social dynamics

#### Subtheme 3a: kindness, unity and acceptance

Participants reported a generally improved culture at medical school. They felt that their peers had been humbled by COVID-19 and the lockdown, bringing them closer together. P2 explained that “I think the lockdown has really brought to our senses what really matters most in the world… So, we have to actually work together and unite to help everybody do their best.” He went on to say that “it has become much more friendly. It has actually brought all of us much closer together, the whole experience.” This sense of kindness and acceptance also extended upwards, with wholly warm feelings towards the medical school staff. P5 explained that, despite things not being perfect, “I definitely don’t blame the medical school for anything they’ve done. They’ve put so much work in.”

#### Subtheme 3b: growing apart

One participant felt that the lack of face-to-face communication was a double-edged sword. Whilst some people had been humbled by the experience, P3 felt that some classmates were crueller and more controlling of others. He explained that “there was quite a bit of hostility on group chats and stuff as well. Saying things like… ‘people should be learning about this—they should be reading about this. Everyone who’s not commenting or liking this—we’ll know who you are’.” This induced a sense of anxiety if he missed any such group messages for any extended periods of time—fearing ‘naming and shaming’ for having not responded—a new form of bullying.

#### Subtheme 3c: seeing others—a frame of reference

Participants reported mixed views on a lack of social interaction. Some, like P1, found this quite challenging: “[Friends] really helped me. We have done sort of lessons for each other in the past, and that we obviously couldn’t do this term, so that I think was a bit of a shame.” P1 also found that this impacted on her self-confidence: “In terms of comparing… you don’t know what everyone is doing—you don’t know what level you are at… [Where] you are on your own, I think sometimes it’s easy to get in your head a bit and think ‘oh no, it’s just me that doesn’t understand it’.” However, P3 felt differently. He explained that “because I… know that I process things slightly differently, I tend not to talk to my peers about that kind of stuff, as I know it just sort of gets me down.”

### Theme 4: worries and wonderings

#### Subtheme 4a: venturing into the unknown

Participants were nervous about the future of their education. P1 felt that “it’s just made me slightly apprehensive for next year… When we are back to normal… you know, keeping up… not being able to go at my own pace.” Anxious feelings were particularly prevalent in those within, or about to enter, clinical years. For example, P2 said that “when it comes to [entering the clinical years after the summer], I am really not sure how it is supposed to go… So, I’m just worried—will our learning be compromised?”.

#### Subtheme 4b: lack of clinical exposure

All participants acknowledged a lack of clinical exposure in their recent training. “I had a lot of my patient-facing (clinical) placements this term which we have just missed… There was nothing to replace my missed GP placements… I just feel sad we missed it and I'm gutted” (P1). P5 was particularly worried about this. She explained that “we were supposed to be getting a 1-week experience in cardiology… That’s where a lot of people really learn a lot about how to read ECGs… I just need to spend a lot more time reading them than other people probably will. It makes me question about how ready I am as an F1, and that scares me.” She also worried that “the skill of just being able to look at a patient and say ‘are they sick? Am I worried about them?’ is something that you can only get through experience.”

Some considered volunteering in hospitals to gain clinical experience. This, however, proved impossible for all. For some, logistics prevented this. “[I] signed up to do it, but… the Trust has been incredibly slow at getting paperwork through, so I still haven't actually been” (P4). For others, the health of them or their loved ones required them to stay home: “I really do want to volunteer… but I’ve got asthma” (P5).

#### Subtheme 4c: clinical assessments, ranking and cheating

All discussed the cancellation of practical assessments due to COVID-19. “Our OSCE was cancelled. I would have quite liked that to go ahead—for my peace of mind over the summer—because I don’t feel ready to do anything we did in it really” (P3). P5 was particularly worried, as her dyslexia made her struggle with written exams. Therefore, due to the lack of practical exams, her class ranking could be disastrously affected. “For our ranking, the only thing that matters is the, erm, [non-clinical written exam] and the essays. It’s terrible, and I’ve lost so much sleep over it… I’m terrible at essays—I always have been. I just hate them” (P5). She went on to explain that “with the [clinical written exam]… it’s a lot harder to cheat… if there’s a chest x-ray you can’t Google what it is… And also, where the [clinical written exam] didn’t really matter other than passing, I don’t think anybody’s going to cheat… But with the [non-clinical written exam], it was literally asking you questions that you could Google in a heartbeat, and it’s going to count towards the majority of… your ranking… Me and my partner had a lot of conversations about [it]… It was really gnawing on the back of our minds that so many people that we know probably would cheat, and that there’s no way we could prove it.” P4 also believed that “there has been rife cheating.”

#### Subtheme 4d: technological issues

Participants also worried about various issues with technology that might hinder their learning or exam performance. Some flagged poor internet connections as a concern. “During the last exam, a lot of students couldn’t do the exam because their Wi-Fi crashed” (P1). Others mentioned worries about the online platforms inadvertently rendering their usual coping strategies inert. “I’m having to really actively listen in a different way than I normally have to, because I don’t have the benefit of lip reading at the moment… If we’ve got, say, a massive class, a lot of people don’t show their faces—just because otherwise the internet quality is so bad.” (P5). There were also concerns over the accessibility of some aspects of their exams. “The [multiple choice exams] worked quite well, but the short answer questions were awful ‘cos the textbox was tiny. You could only see 3 or 4 words and you obviously have to write a few sentences” (P4).

## Discussion

This study has explored the learning experiences of dyslexic medical students during the COVID-19 pandemic. Our participants found learning and studying during this time to be a largely positive experience. Generally, it fostered a culture of togetherness and allowed them greater flexibility in their learning—giving them the opportunity to overcome the difficulties associated with their dyslexia. However, as P3 reported, he felt that some classmates were crueller and more controlling of others—the “double-edged sword”. Cyber-bullying had replaced person to person bullying,

Interestingly, our e-learning findings are at odds with much of the wider literature, which suggest that e-learning may disadvantage dyslexic students (Woodfine et al., 2008). Our results are more aligned with those of Newlands et al., who found that dyslexic junior doctors made use of various technologies to better engage in their jobs (Newlands et al., 2015). It has been shown that dyslexic HE students are less likely than their peers to be organised in their approach to studying when distance learning (Jelfs & Richardson, 2010). These students have also been found to achieve lower academic attainment when using distance learning approaches compared to their non-dyslexic peers (Jelfs & Richardson, 2010). This is in keeping with Debenham, who reported that disabled students had to put in more effort than their non-disabled peers in order to achieve comparable outcomes (Debenham, 2001). Despite this, e-learning and distance learning can have many positive points for dyslexic learners. For example, through the ease of access to text-to-speech software and embedded grammar software (Alsobhi et al., 2015). However, such approaches can present a double-edged sword. It has been argued that chat software and video conferencing can put dyslexic students at a disadvantage (Woodfine et al., 2008). It has also been reported that dyslexic HE students may be less likely to pass online modules and may achieve lower grades compared to their non-dyslexic peers (Richardson, 2015). We were unable to locate any data concerning this involving medical students. Interestingly, however, Richardson also reported that dyslexic students were more likely to undertake courses involving health and social care (Richardson, 2015). Many accessibility guidelines within educational settings are also based on reading and writing and not e-learning communication tools (Pang & Jen, 2017). It has previously been reported that text-based chat systems can put excess strain on learners with SpLDs (Pang & Jen, 2017). This could be an important consideration in the review and design of e-learning for medical students during the COVID-19 pandemic. At this point, it should also be noted that many of the existing studies are limited due accessibility standards throughout the educational sector for e-learning being considered subpar (Cinquin et al., 2019). Furthermore, none of these studies have focused on medical students. There is therefore a need for further research in this area.

During our interviews it became clear that several participants felt their colleagues had cheated during the remote, online exams. This raised potential professionalism concerns for the students involved. Within a vocational profession such as medicine, where patient safety and fitness to practice are paramount, such behavior should be viewed with caution—especially when we know that “unprofessional behaviour in medical school is associated with later unprofessional behaviour by practitioners” (Tonkin, 2015). “Cheating in exams damages the validity of assessment and the standing of the medical profession” (Tonkin, 2015). However, the issue of exams during this unprecedented time may be more complex than it first appears. The dramatic restructuring of exams became necessary due to national lockdowns as well as social distancing needs (Jervis & Brown, 2020). However, there were several concerns for students and teaching staff, including safe spaces, possible caring commitments for other household members, and poor or non-existent internet access (Jervis & Brown, 2020). Institutions tried to mitigate these concerns in a variety of ways—by increasing time for exams, or by using software that automatically saved answers as students progressed through the exams, for example (Mathieson et al., 2020).

The positive change in medical school culture to one of support and unity is both interesting and reassuring. Previous studies have highlighted a toxic, competitive culture, driven by the ranking of students against their peers (Dennis et al., 2012). This has been highlighted as a reason that medical students may not want to admit to struggling—through fear of it being seen as weakness by their peers (Dennis et al., 2012). Humayon & Soaib described medical school as an environment in which “survival of the fittest is the ultimate goal” and where “competition holds the utmost significance" (Humayon & Shoaib, 2019). Previous research has highlighted similar issues specific to dyslexic medical students. For example, experiences of bullying, isolation, and a fear of stigmatization (Anderson & Shaw, 2020; Shaw & Anderson, 2018b). It seems that the experience of the COVID-19 lockdown may have helped to improve these issues in the eyes of our participants. Further, more specific research in this area may yield interesting findings.

The increased control over their education reported by our participants is another positive finding. Studies have shown that taking control of one’s learning is highly beneficial to the learning process and that active learning, where individuals actively engage with study, is superior to passive learning, where they absorb information from teaching—such as lectures—without engaging with or seeking meaning in the content (Shaw, 2017). Furthermore, self-regulated learning is a vital aspect of life-long learning and “self-regulated learners are more effective in learning and have a repertoire of learning and study strategies to match different situations” (Jouhari et al., 2016). Active, self-regulated learning therefore becomes more important as students progress to the later stages of their training (Shaw, 2017). Therefore, the COVID-19 pandemic may yet have the unexpected outcome of improving our students’ lifelong, self-regulated learning abilities. This control also extended to the manipulation of the learning materials to best suit their preferences and needs.

The adapted teaching approaches used during the pandemic were applauded by our participants. They expressed a wish for these to continue once their medical school resumes face-to-face teaching. However, research is needed to ascertain which teaching and assessment methods may have been most effective before a long-term change in delivery is recommended. For example, whilst we know that multiple choice exams are fair for dyslexic medical students (Ricketts et al., 2010), we do not know if this remains the case when undertaken at home on computers.

Our results did not find that the pandemic had induced helpless or hopeless states in our participants—quite the reverse. Given that this potential has been highlighted previously (Shaw, 2020), this is a positive finding. Our participants actually found that their stress levels were reduced, and they had far greater control over their education—a situation that is inherently at odds with the development of helplessness. Their positive responses to the pandemic may be the result of training in resilience at undergraduate level. It is, however, also possible that removal from the aforementioned competitive environment may have played a protective role. Further research is now needed to explore this.

Some participants demonstrated self-deprecating feelings through their reports, which did not reflect a view of dyslexia in keeping with neurodiversity or the social model of disability. For example, P5’s comment that “I definitely don’t think that there’s a scenario in which everybody comes out of this happy with COVID. Because, if you’re being fair to me, you’re putting somebody else at a disadvantage.” It would be remiss of us to not consider possible deeper meaning here. This comment emphasises equality over equity and may suggest a personal sense of guilt at needing support that non-dyslexic peers do not. Our previous work has identified that a minority of medical students do resent the support given to their dyslexic colleagues (Hennessy et al., 2020). This has previously led to frustration, negative comments and bullying (Hennessy et al., 2020). P5’s concern here may reflect on-going issues in this area.

### Study strengths and limitations

Here we have presented the first study exploring the experiences of dyslexic medical students in relation to the COVID-19 pandemic. We have also reported the first data concerning their experiences in relation to distance learning, e-learning, and remote exams. This study provides a unique and important window into their World during these unprecedented times. Our (SS and LH) insider status as dyslexic doctors would also be considered a strength within our interpretive phenomenological research design.

Our participants being few in number and coming from a single medical school is both a strength and a potential weakness. Phenomenological research works best with small numbers of homogeneous participants, given its aim to explore a shared experience in depth. However, our results should not be considered generalizable in the traditional, positivist sense. This is not in itself considered a weakness to such studies, however, as phenomenological research does not aim to produce generalizable findings—rather they offer reports of people’s experiences in the hope that we can learn from those, i.e. transferability.

It is also important to consider our participants themselves. Those who participate in projects such as this may choose to do so because they hold particularly strong views or experiences that they wish to express. Participants are also likely to only take part if they feel they have adequate time to do so. It is possible that this could exclude those who are struggling more with workloads during this challenging time. This might have led to some of our more positive findings.

## Conclusions

Here we have explored the learning experiences of dyslexic medical students during the COVID-19 pandemic. Our results highlighted a largely positive experience, with an improved culture of togetherness, improved freedom and control. They also revealed issues with a lack of clinical exposure, potential negative impacts on ranking positions, and suspected cheating in exams. It is our hope that medical educators may resist an automatic return to ‘the way things have always been done’ when the pandemic has resolved. Rather, we need to consider what lessons can be learnt from this situation, and by doing so, continue to foster a more positive culture and paradigm shift within medical schools. For example, moving forwards, medical schools might consider flexibility in timetabling through the on-going use of blended learning. This may allow dyslexic students to take better control of their own learning, in their preferred environments, at their own pace. This could be further facilitated through the provision of lecture recordings and teaching materials that students can review at their own pace.

Further research is needed to quantify our findings and to explore the various issues we have highlighted in more detail. It is our hope that others, with greater resources than we had available to us for this study, will take advantage of the quasi-experimental situation afforded by the impact of the COVID pandemic, to research in more detail the effects of a greater shift to remote learning in medical education.

## References

[CR1] Ahmed S, Jafri L, Majid H, Khan AH, Ghani F, Siddiqui I (2020). Challenges amid covid-19 times - review of the changing practices in a clinical chemistry laboratory from a developing country. Annals of Medicine and Surgery..

[CR2] Alsobhi AY, Khan N, Rahanu H (2015). Dael framework: A new adaptive e-learning framework for students with dyslexia. Procedia Computer Science..

[CR3] Anderson JL, Shaw SCK (2020). The experiences of medical students and junior doctors with dyslexia: A survey study. International Journal of Social Sciences and Educational Studies..

[CR4] Britsh Medical Association. Widening participation in medicine. 2020. [accessed 2020 Jun 14]. https://www.bma.org.uk/advice-and-support/studying-medicine/becoming-a-doctor/widening-participation-in-medicine.

[CR5] Britain G (2010). Equality act 2010.

[CR6] Cinquin P, Guitton P, Sauzeon H (2019). Online e-learning and cognitive disabilities: A systematic review. Computers & Education..

[CR7] Clouder L, Karakus M, Cinotti A, Ferreyra MV, Fierros GA, Rojo P (2020). Neurodiversity in higher education: A narrative synthesis. Higher Education..

[CR8] Connell-Smith A, Hubble S. 2018. Widening participation strategy in higher education in england. United Kingdom.

[CR9] Debenham M. 2001. Computer mediated communication and disability support: Addressing barriers to study for undergraduate distance learners with long-term health problems. [United Kingdom]: The Open University.

[CR10] Dennis A, Warren R, Neville F, Laidlaw A, Ozakinci G (2012). Anxiety about anxiety in medical undergraduates. Clinical Teacher..

[CR11] Dyslexia International. The problem. n.d. [accessed 2017 Jan 21]. http://www.dyslexia-international.org/the-problem/.

[CR13] Goodley D, Lawthom R, Liddiard K, Runswick-Cole K (2019). Provocations for critical disability studies. Disability & Society..

[CR14] Hennessy L, Shaw S, Anderson J (2020). Medical students’ attitudes towards and beliefs about dyslexia: A single-centre survey study. International Journal of Social Sciences & Educational Studies..

[CR15] Holland F (2014). Teaching in higher education: An interpretive phenomenological analysis. Sage research methods cases.

[CR16] Humayon Z, Shoaib S (2019). Relationship of competitiveness, jealousy, disgust and envy among medical students. Bahria University Journal of Humanities and Social Sciences..

[CR17] Jelfs A, Richardson J (2010). Perceptions of academic quality and approaches to studying among disabled and nondisabled students in distance education. Studies in Higher Education..

[CR18] Jervis C, Brown L (2020). The prospects of sitting ‘end of year’ open book exams in the light of covid-19: A medical student’s perspective. Medical Teacher..

[CR19] Jouhari Z, Haghani F, Changiz T (2016). Assessment of medical students' learning and study strategies in self-regulated learning. Journal of Advances in Medical Education & Professionalism.

[CR20] Lopez KA, Willis DG (2004). Descriptive versus interpretive phenomenology: Their contributions to nursing knowledge. Qualitative Health Research.

[CR21] Mackey S (2005). Phenomenological nursing research: Methodological insights derived from heidegger's interpretive phenomenology. International Journal of Nursing Studies.

[CR22] Madriaga M (2007). Enduring disablism: Students with dyslexia and their pathways into uk higher education and beyond. Disability & Society..

[CR23] Mathieson G, Sutthakorn R, Thomas O (2020). Could the future of medical school examinations be open-book - a medical student's perspective?. Medical Education Online..

[CR24] Medical Schools Council. 2018. Selection alliance 2018 report: Update on the medical schools council's work in selection and widening participation. United Kingdom.

[CR25] Musto JS. 2013. How do medical students with specific learning difficulties (spld) cope in a clinical setting? Chapter 1. Norwich Medical School.

[CR26] Nelson RH. 2020. A critique of the neurodiversity view. Journal of Applied Philosophy. Online ahead of print.

[CR27] Newlands F, Shrewsbury D, Robson J (2015). Foundation doctors and dyslexia: A qualitative study of their experiences and coping strategies. Postgraduate Medical Journal.

[CR28] Ng YM, Or PLP (2020). Coronavirus disease (covid-19) prevention: Virtual classroom education for hand hygiene. Nurse Education in Practice..

[CR29] Oliver M (2013). The social model of disability: Thirty years on. Disability & Society..

[CR30] Pang L, Jen CC (2017). Inclusive dyslexia-friendly collaborative online learning environment: Malaysia case study. Education and Information Technologies..

[CR31] Pietkiewicz I, Smith JA (2014). A practical guide to using interpretive phenomenological analysis in qualitative research psychology. Czasopismo Psychologiczne Psychological Journal..

[CR32] Reaume G (2014). Understanding critical disability studies. Canadian Medical Association Journal..

[CR33] Richardson JTE (2015). Academic attainment in students with dyslexia in distance education. Dyslexia.

[CR34] Ricketts C, Brice J, Coombes L (2010). Are multiple choice tests fair to medical students with specific learning disabilities?. Advances in Health Sciences Education: Theory and Practice.

[CR35] Shakespeare T, Watson N. 2015. The social model of disability: An outdated ideology? Exploring theories and expanding methodologies: Where we are and where we need to go. p. 9–28.

[CR36] Shaw SCK, Malik M, Anderson JL. 2017. The exam performance of medical students with dyslexia: A review of the literature. MedEdPublish. 6(3). 10.15694/mep.2017.000116 PMC10885271 38406421

[CR37] Shaw SCK, Grant AJ, Anderson JL. 2018. Autoethnography in action: A research methods case study on the use of a collaborative autoethnography to explore the culture of studying medicine with dyslexia. In SAGE Research Methods Cases.

[CR38] Shaw SCK, Anderson JL. 2018a. Phenomenological research in medical education: An overview of its philosophy, approaches and conduct. In SAGE Research Methods Cases.

[CR39] Shaw SC (2017). How can we promote and facilitate effective study skills in medical students?. MedEdPublish..

[CR40] Shaw SCK (2018). Learned helplessness in doctors with dyslexia: Time for a change in discourse?. Nurse Education in Practice..

[CR41] Shaw SCK (2019). An introduction to the role of relational ethics in qualitative healthcare education research. Nurse Education in Practice..

[CR42] Shaw SCK (2020). Hopelessness, helplessness and resilience: The importance of safeguarding our trainees' mental t wellbeing during the covid-19 pandemic. Nurse Education in Practice..

[CR43] Shaw SCK, Anderson JL (2017). Twelve tips for teaching medical students with dyslexia. Medical Teacher.

[CR44] Shaw SCK, Anderson JL (2018). The experiences of medical students with dyslexia: An interpretive phenomenological study. Dyslexia.

[CR45] Shaw SCK, Anderson JL, Grant AJ (2016). Studying medicine with dyslexia: A collaborative autoethnography. Qual Rep..

[CR46] Shaw SCK, Hennessy LR, Okorie M, Anderson JL (2019). Safe and effective prescribing with dyslexia. BMC Medical Education..

[CR47] Tonkin A. 2015. "Lifting the carpet" On cheating in medical school exams. BMJ. 351:h4014. 10.1136/bmj.h4014 26286467

[CR48] Walker ER, Shaw SCK (2018). Specific learning difficulties in healthcare education: The meaning in the nomenclature. Nurse Education in Practice..

[CR49] Walker ER, Shaw SCK, Anderson JL (2020). Dyspraxia in medical education: A collaborative autoethnography. The Qualitative Report..

[CR50] Walker ER, Shaw SC, Price J, Reed M, Anderson J (2018). Dyspraxia in clinical education: A review. The Clinical Teacher..

[CR51] Walker ER, Shaw SCK, Reed M, Anderson JL (2021). The experiences of foundation doctors with dyspraxia: A phenomenological study. Advances in Health Sciences Education: Theory and Practice.

[CR52] Woodfine BP, Bapista Nunes M, Wright DJ (2008). Text-based synchronous e-learning and dyslexia: Not necessarily the perfect match!. Computers & Education..

